# Educational attainment among primary school children with neurodisability: a population-based cohort study using linked education and health data from England

**DOI:** 10.1136/archdischild-2025-329224

**Published:** 2026-03-22

**Authors:** Ayana Cant, Ania Zylbersztejn, Laura Gimeno, Vincent Nguyen, Joachim Tan, Ruth Gilbert, Katie L Harron, on behalf of the HOPE Study Group, Katie Harron

**Affiliations:** 1 Department of Population, Policy and Practice, UCL GOS Institute of Child Health, London, UK; 2 NIHR Great Ormond Street Hospital Biomedical Research Centre, London, UK; 3 Great Ormond Street Institute of Child Health, University College London, London, UK; 4 Social Research Institute, Centre for Longitudinal Studies, University College London, London, UK; 5 Institute for Health Informatics, University College London, London, UK

**Keywords:** Child Health, Neurology, Paediatrics, Epidemiology

## Abstract

**Objective:**

To compare educational attainment between children with and without hospital-recorded neurodisability in England at ages 5, 7 and 11 using linked administrative hospital and education data in the Education and Child Health Insights from Linked Data (ECHILD) database.

**Design:**

Population-based cohort study.

**Methods:**

We derived a national birth cohort of 2 351 589 children born in England between 1 September 2003 and 31 August 2008 enrolled in state-funded primary schools in Reception (age 4–5) using linked health and education records. Neurodisability, defined here as chronic conditions affecting the nervous system which result in functional limitations, is identified using hospital admission records. We described differences in primary school educational attainment for children with and without neurodisability.

**Results:**

About 2.2% of children had a recorded neurodisability before starting Reception. These children consistently underperformed in national assessments, with fewer than half meeting nationally expected levels in Maths and English at every time point (vs ~70% of peers). By the end of primary school (age 10/11), 31% of children with neurodisability (vs 6% of peers) did not participate in national assessments despite being enrolled in school. Among children with neurodisability, educational attainment was lowest for children with Down syndrome and highest for children with perinatal conditions.

**Implications:**

Substantial attainment gaps exist between children with and without neurodisability and persist across primary school. By age 11, many children with neurodisability do not undertake national assessments, highlighting the need for personalised, functional outcome measures to capture their educational development.

**WHAT IS ALREADY KNOWN ON THIS TOPIC**

- Children with neurodisability face functional challenges that can disrupt their success in school.
- There is limited understanding of their participation in national assessments and academic attainment throughout primary school on a population level in England.

**WHAT THIS STUDY ADDS**

- Longitudinal analysis of whole-population cohorts from ages 4 to 11 found that attainment gaps between children with and without neurodisability remained largely constant across ages. Seven out of 10 children with neurodisability who enter Year 1 are not ‘school ready’ at age 5 (vs 40% of peers). Despite being enrolled in school at age 11, one in three children with neurodisability did not participate in assessments.

**HOW THIS STUDY MIGHT AFFECT RESEARCH, PRACTICE OR POLICY**

- Hospital records can identify, before school entry, children with neurodisability at greater risk of poorer educational outcomes. Current attainment measures do not fully capture the educational development of these children, highlighting the need for more personalised outcome measures to inform meaningful population-level research and policy evaluations.

## Introduction

 Education aims to promote the socioemotional, cognitive and physical potential of every child. However, chronic health conditions in childhood can disrupt educational experiences, placing affected children at risk of poorer outcomes in later life.^1^ As a social determinant of lifelong outcomes, education must be prioritised for children with chronic conditions to promote their health, well-being and societal participation.

Neurodisability refers to a set of chronic conditions attributed to impairments of the nervous system, leading to functional limitations in movement, cognition, hearing, vision, communication, emotion or behaviour.^2^ Children with neurodisability often face complex healthcare and educational needs. Neurodisability includes neurodevelopmental disorders (eg, attention-deficit/hyperactivity disorder (ADHD), autism spectrum disorder (ASD), specific learning disabilities such as dyslexia, dyspraxia and dyscalculia), neurological disorders (eg, epilepsy, cerebral palsy), as well as a broader range of genetic, muscular and perinatal conditions that affect brain development and learning.^1^ While individual conditions are rare, neurodisability affects around 1 (3.6%) in 25 primary school children born in England collectively.^3^

Population cohort studies have been conducted internationally (eg, Scotland,^4 5^ Wales,^6^ Australia^7^) and indicate that neurodisability is associated with lower academic performance, higher school absenteeism and more need for additional learning support than unaffected peers.^4–8^ Attainment disparities arise from barriers to participation, including health-related absences, physical access challenges, communication difficulties, cognitive impairments and emotional or behavioural difficulties.^9–11^ Existing research typically examines individual neurodisability conditions in isolation, with limited population-level evidence on educational attainment across the wider school-aged neurodisability population in England.

Quantifying attainment differences between children with neurodisability and their peers provides prognostic insight for those participating in standard assessments and those not formally assessed, supporting local and national education planning and coordinated health–education provision.

In this study, we used the Education and Child Health Insights from Linked Data (ECHILD) database^12^ of linked education and hospital records for all children in England to describe educational attainment throughout primary school at ages 5, 7 and 11 for children with neurodisability compared with their peers, and for five subgroups of neurodisability (neurodevelopmental disorders, epilepsy, cerebral palsy, Down syndrome and perinatal conditions).

## Methods

A protocol for this study was published in March 2024 (online supplemental material A).^13^

### Participants

We used the ECHILD database which primarily links administrative data from the National Pupil Database (NPD) and Hospital Episode Statistics (HES).^12^ The version of ECHILD used contained data on approximately 14.7 million children and young people aged 0–24 in England who were born between 1 September 1995 and 31 August 2020.^13^ Education data from the NPD contains pupil-level information on registration, attainment, exclusions and absences for children attending state-funded schools in England from 2001/2002 onwards.^14^ HES records all hospital admissions in National Health Service (NHS)-funded hospitals in England (including birth), capturing 98%–99% of all hospitalisations.^15^ Records were deterministically linked by NHS England.^14^

The study included all singleton children born in NHS-funded hospitals between 1 September 2003 and 31 August 2008 linked to the NPD (online supplemental figure S1). This period ensured that all children completed primary school before the COVID-19 pandemic disrupted attendance, learning and assessments.^16^ Children were included if they appeared in the Spring School Census of Reception (age 4/5) at a state-funded school. Children were followed from Reception until the end of Year 6 (age 10/11) or death, whichever occurred first (online supplemental figure S2). As the dataset covers approximately 93% of children in England attending state schools,^17^ findings are broadly population-representative, though they may not capture the experiences of the small minority in private schools who are on average more socioeconomically advantaged. Cohort derivation details are included in the online supplemental material B.

The research project was informed by children, young people, parent-carers and professional groups to understand their experiences of school and views on the use of linked health, education and social care data for research. In focus groups and interviews, participants emphasised the importance of including the whole population to investigate the inter-related areas of health and education.^17^ Exams and performance under the National Curriculum at school were points of stress for children with health conditions, forming a key outcome measure in this study. Key learnings from past public engagements can be found at: https://www.echild.ac.uk/engaging-with-the-public.

### Exposure: hospital-recorded neurodisability

Neurodisability was identified using relevant diagnostic or procedure codes recorded in hospital admissions or as a cause of death between birth and the start of Reception, ensuring exposure preceded educational outcomes. Children without relevant codes were classified as peers without neurodisability, though some may have been diagnosed later. The clinically developed and validated code list is published in the ECHILD phenotyping library.^3 18^ Codes represent conditions where ≥50% of affected children are expected to have neurological impairment or functional limitations, acknowledging under-recording in hospital data. Approximately two-thirds of children with hospital-recorded neurodisability were identified from codes documented before age 5. Common conditions included perinatal disorders (eg, brain injury, extreme prematurity), congenital or inherited anomalies, developmental disorders, epilepsy and cerebral palsy. These informed subgroup analyses. Subgroups were not mutually exclusive due to diagnostic overlap.

### Outcome: primary school attainment

We assessed attainment at the end of Reception, termed the Early Years Foundation Stage Profile (EYFSP, age 4/5), at Key Stage 1 in Year 2 (KS1, age 6/7) and at Key Stage 2 in Year 6 (KS2, age 10/11) using national assessments. At each Key Stage, we derived (1) the proportion of children who did not complete the assessment to be taken in that year and of those who did; (2) the proportion of children who reached nationally expected levels in these assessments as defined in line with Department of Education standards; and (3) the cohort-specific standardised test scores calculated using the mean and SD of all children’s scores who were assessed in a given academic year. We also examined the EYFSP ‘Good Level of Development’ (GLD) measure, indicating school readiness across key learning domains.^19^ Full outcome derivation is detailed in online supplemental table S1.

### Statistical analysis

We described cohort derivation, neurodisability prevalence at school entry and demographic characteristics by neurodisability status, including sex, deprivation (Income Deprivation Affecting Children Index (IDACI) quintiles), free school meal (FSM) eligibility, ethnicity, month of birth, region, maternal age, gestational age and birth weight (online supplemental table S2).

For each assessment stage (Reception, Year 2, Year 6), we reported proportions of children who were not assessed, assessed but did not meet nationally expected levels or assessed and achieved expected levels, and calculated mean cohort-standardised Maths and English scores among assessed children. We also described special educational needs and disability (SEND) provision (SEN Support and education, health and care plan (EHCP)) across school years and settings (mainstream/special).

Relative differences in attainment were estimated using Poisson regression with robust standard errors to derive risk ratios for not meeting expected levels (including non-assessment). Models were unadjusted and adjusted for birth season, birth year, deprivation, region, ethnicity and FSM eligibility, guided by a Directed Acyclic Graph (online supplemental figure S3). Analyses were stratified by sex and prematurity to assess effect modification.

### Secondary analyses

Given the heterogeneity and complexity within the neurodisability population, we conducted subgroup analyses for common conditions recorded in HES: (1) neurodevelopmental disorders (ASD, learning disability, developmental disorders); (2) cerebral palsy; (3) epilepsy; (4) Down syndrome; and (5) perinatal conditions (extreme prematurity, extremely low birth weight, perinatal brain injury). We described the prevalence of each subgroup across birth cohorts; mortality rates are reported in online supplemental table S3.

To account for increasing non-participation in assessments over time, we performed a sensitivity analysis restricted to children who completed all primary school assessments. We compared sociodemographic characteristics between children with and without neurodisability within this subgroup to assess potential selection bias.

## Results

Of 2 351 589 pupils included in the study cohort, 51 289 (2.2%) had a hospital record indicating neurodisability before starting Reception (table 1). Children with neurodisability had a higher mortality rate before the end of primary school (0.91% vs 0.03% in children without neurodisabilities; online supplemental table S4). Children with neurodisability were also more likely to be assigned male sex at birth, being Reception at age 5 instead of age 4, attend special school, be eligible for free school meals, live in more deprived neighbourhoods and be born prematurely (<37 weeks gestation) or with low birth weight (<2500 g; online supplemental table S5).

**Table 1:** Prevalence of neurodisability and subtype conditions by academic year of birth

Academic year of birth	Total children (N)	Any ND n (%)	NDD n (%)	Cerebral palsy n (%)	Epilepsy n (%)	Down syndrome n (%)	Perinatal conditions n (%)
2003/2004	435 678	9326 (2.14%)	1309 (0.30%)	891 (0.20%)	1349 (0.31%)	436 (0.10%)	3439 (0.79%)
2004/2005	460 791	9716 (2.11%)	1443 (0.31%)	891 (0.20%)	1410 (0.31%)	498 (0.11%)	3374 (0.73%)
2005/2006	475 766	10 227 (2.15%)	1728 (0.36%)	876 (0.18%)	1427 (0.30%)	482 (0.10%)	3521 (0.74%)
2006/2007	486 966	10 644 (2.19%)	1984 (0.41%)	932 (0.19%)	1458 (0.30%)	526 (0.11%)	3664 (0.75%)
2007/2008	492 388	11 376 (2.31%)	2409 (0.49%)	924 (0.19%)	1560 (0.32%)	440 (0.09%)	3759 (0.76%)
Overall cohort	2 351 589	51 289 (2.18%)	8873 (0.37%)	4514 (0.19%)	7204 (0.31%)	2382 (0.10%)	17 757 (0.76%)

ND refers to neurodisability recorded in hospital admissions before school start (age 4/5).

ND, neurodisability; NDD, neurodevelopmental disorders.

### SEND provision

Children with neurodisability were consistently more likely to receive SEND provision across all time points and levels of intensity (table 2). Over half received some form of provision across Reception, KS1 and KS2 (vs ~18% of peers) and over a quarter had a record of an EHCP at each timepoint (vs 0.2%–2% of peers). By Year 6, 34% of children with neurodisability had an EHCP, compared with only 2% of their peers.

**Table 2:** Educational outcomes across primary school by hospital-recorded ND status

	No ND before school n (%)	ND before school n (%)
Total	2 300 300 (100%)	51 289 (100%)
EYFSP		
SEND provision		
SEN support	249 683 (10.9%)	14 502 (28.3%)
EHCP (mainstream school)	12 475 (0.5%)	7468 (14.6%)
EHCP (special school)	4589 (0.2%)	5574 (10.9%)
Any SEND provision	420 723 (18.3%)	26 118 (50.9%)
Good level of development		
Achieving expected level	1 316 213 (57.2%)	15 290 (29.8%)
Not achieving expected level	968 942 (42.1%)	35 220 (68.7%)
Not assessed	15 145 (0.7%)	779 (1.5%)
English		
Achieving expected level	1 584 145 (68.9%)	20 229 (39.4%)
Not achieving expected level	701 010 (30.5%)	30 281 (59.0%)
Not assessed	15 145 (0.7%)	779 (1.5%)
Standardised assessment score		
Mean (SD)	0.04 (0.96)	−0.92 (1.45)
Maths		
Achieving expected level	1 811 241 (78.7%)	25 616 (49.9%)
Not achieving expected level	473 914 (20.6%)	24 894 (48.5%)
Not assessed	15 145 (0.7%)	779 (1.5%)
Standardised assessment score		
Mean (SD)	0.04 (0.95)	−0.95 (1.61)
Key Stage 1		
SEND provision		
SEN support	399 104 (17.4%)	16 169 (31.5%)
EHCP (mainstream school)	18 173 (0.8%)	8420 (16.4%)
EHCP (special school)	6029 (0.3%)	6097 (11.9%)
Any SEND provision	419 346 (18.2%)	29 915 (58.3%)
English		
Achieving expected level	1 526 372 (66.4%)	19 125 (37.3%)
Not achieving expected level	725 270 (31.5%)	30 437 (59.3%)
Not assessed	48 658 (2.1%)	1727 (3.4%)
Standardised assessment score		
Mean (SD)	0.05 (0.91)	−0.93 (1.43)
Maths		
Achieving expected level	1 794 705 (78.0%)	23 998 (46.8%)
Not achieving expected level	455 723 (19.8%)	25 259 (49.2%)
Not assessed	49 872 (2.2%)	2032 (4.0%)
Standardised assessment score		
Mean (SD)	0.05 (0.95)	−1.02 (1.56)
Key Stage 2		
SEND provision		
SEN support	365 660 (15.9%)	11 758 (22.9%)
EHCP (mainstream school)	47 603 (2.1%)	10 284 (20.1%)
EHCP (special school)	14 144 (0.6%)	7253 (14.1%)
Any SEND provision	420 723 (18.3%)	28 843 (56.2%)
English		
Achieving expected level	1 690 039 (73.5%)	23 399 (45.6%)
Not achieving expected level	463 019 (20.1%)	11 984 (23.4%)
Not assessed	147 242 (6.4%)	15 906 (31.0%)
Standardised assessment score		
Mean (SD)	0.03 (0.98)	−0.34 (1.12)
Maths		
Achieving expected level	1 724 372 (75.0%)	23 500 (45.8%)
Not achieving expected level	429 608 (18.7%)	11 902 (23.2%)
Not assessed	146 320 (6.4%)	15 887 (31.0%)
Standardised assessment score		
Mean (SD)	0.01 (0.99)	−0.41 (1.16)

EHCP, education, health and care plan; EYFSP, Early Years Foundation Stage Profile; ND, neurodisability; SEND, special educational needs and disability.

### Achieving expected levels in national assessments

A large proportion of children with neurodisability failed to reach expected attainment levels in every primary school assessment. Achievement rates for children with neurodisability varied from the lowest observed rate in the EYFSP assessments in Reception, where 30% met the GLD standard (vs 57% of peers), to the highest observed achievement in the Reception Maths assessment, where 50% of children with neurodisability achieved expected levels (vs 79% of peers; table 2).

**Figure 1:**
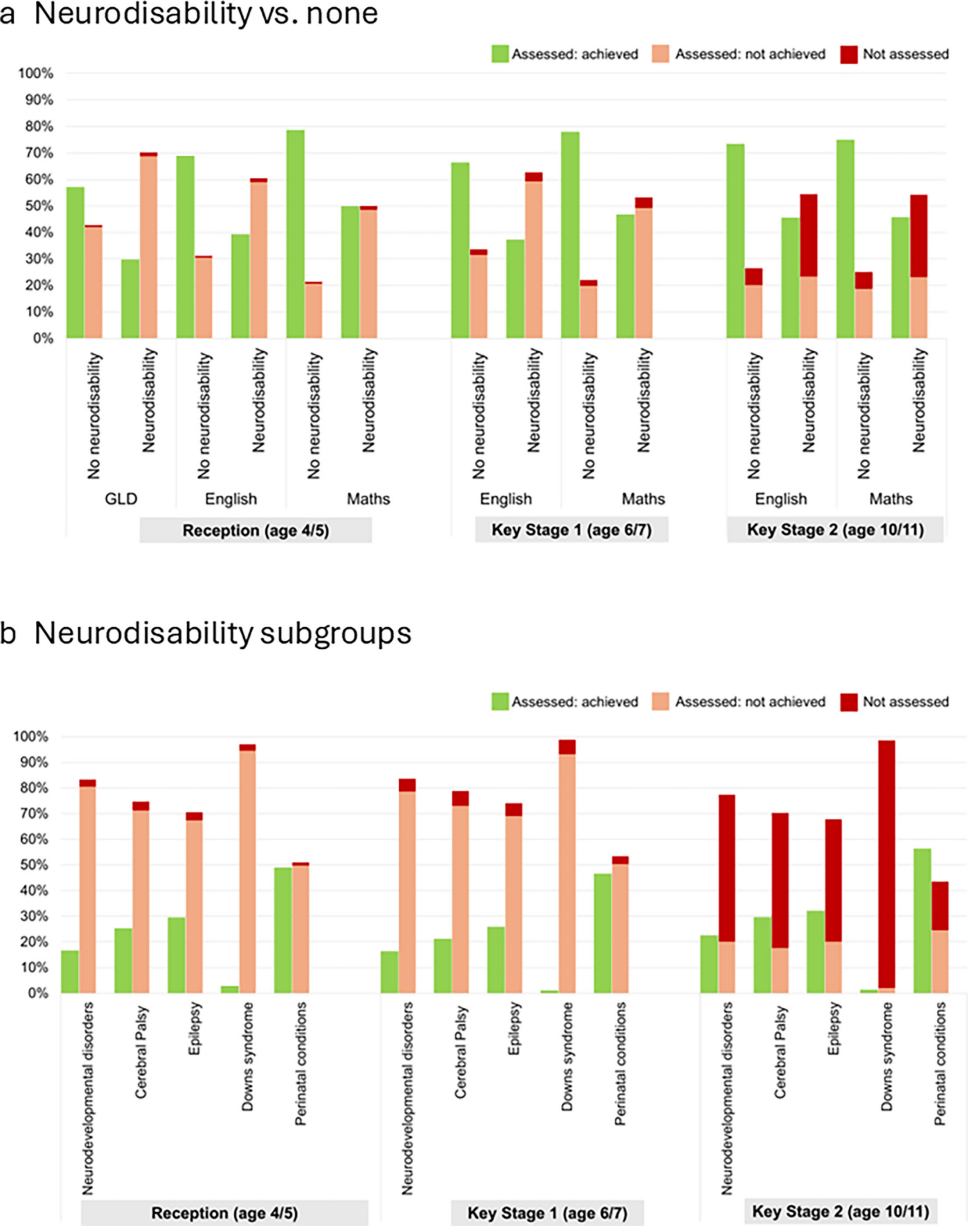
Proportion of children achieving nationally expected levels in primary school assessments, by hospital-recorded neurodisability (ND) status and subgroup. Figure 1a compares children with any hospital-recorded neurodisability to those with no recorded neurodisability. Figure 1b compares outcomes across neurodisability subgroups. Neurodisability status and subgroups are defined based on hospital records prior to the start of Reception (age 4–5). Outcomes include achievement at the Early Years Foundation Stage Profile (EYFSP), Key Stage 1 (KS1), and Key Stage 2 (KS2).

While achievement rates were similar across KS1 and KS2, non-participation in assessments increased substantially. In Reception, 1.5% of children with neurodisability were not assessed under the EYFSP, compared with 0.7% of peers. At KS1, non-assessment rates rose to 3.4% in English and 4.0% in Maths (vs 2.1% and 2.2% in peers). At KS2, non-participation in both Maths and English rose to 31%, compared with 6.4% of unaffected peers, despite continued school enrolment. Among children with neurodisability, the substantial increase in non-participation became the primary driver of failing to reach expected levels in assessments at the end of primary school (figure 1a).

**Figure 2:**
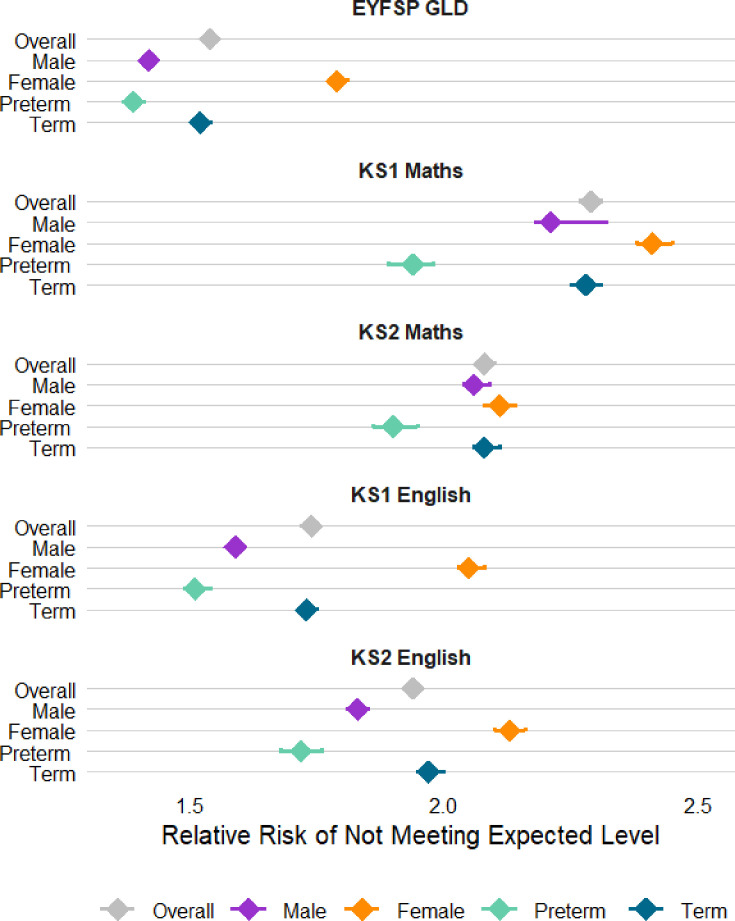
Adjusted risk ratios for not achieving expected levels across primary school assessments comparing children hospital-recorded neurodisability to those without. EYFSP, Early Years Foundation Stage Profile; GLD, Good Level of Development; KS1, Key Stage 1; KS2, Key Stage 2.

Children with neurodisability were more than twice as likely to not achieve expected levels in Maths assessments at all time points (table 3). The adjusted relative risk for not achieving expected levels was 2.16 (95% CI 2.14 to 2.18) at Reception, 2.29 (95% CI 2.27 to 2.31) at KS1 and 2.08 (95% CI 2.07 to 2.10) at KS2. Similarly, children with neurodisability were over 1.7 times more likely to fail English assessments at all three stages compared with their peers. The association between neurodisability and not achieving expected levels in Maths and English was more pronounced among females than males (figure 2). Additionally, relative risks indicated that educational disparities between children with neurodisability and their peers were greater among those born at term than among those born preterm at every time point (table 3, figure 2).

**Table 3:** Unadjusted and adjusted risk ratios for not achieving expected levels in assessments by ND status before school

	Unadjusted	Adjusted
	ND before school RR (95% CI)	ND before school RR (95% CI)
EYFSP		
GLD		
Overall	1.64 (1.63–1.65)	1.54 (1.53–1.55)
Males	1.46 (1.45–1.47)	1.42 (1.41–1.43)
Females	1.87 (1.85–1.89)	1.79 (1.78–1.81)
Preterm (<37 weeks)	1.42 (1.40–1.44)	1.39 (1.37–1.41)
Term (37+ weeks)	1.62 (1.61–1.64)	1.52 (1.51–1.54)
English		
Overall	1.94 (1.93–1.96)	1.79 (1.78–1.81)
Males	1.72 (1.70–1.73)	1.64 (1.63–1.66)
Females	2.26 (2.26–2.28)	2.12 (2.09–2.15)
Preterm (<37 weeks)	1.60 (1.56–1.63)	1.56 (1.53–1.58)
Term (37+ weeks)	1.93 (1.91–1.95)	1.78 (1.76–1.80)
Maths		
Overall	2.35 (2.33–2.38)	2.16 (2.14–2.18)
Males	2.17 (2.14–2.19)	2.04 (2.01–2.06)
Females	2.58 (2.53–2.61)	2.39 (2.35–2.43)
Preterm (<37 weeks)	1.86 (1.81–1.91)	1.79 (1.75–1.83)
Term (37+ weeks)	2.34 (2.31–2.38)	2.16 (2.13–2.19)
Key Stage 1		
English		
Overall	1.86 (1.85–1.88)	1.74 (1.72–1.75)
Males	1.65 (1.63–1.66)	1.59 (1.58–1.60)
Females	2.16 (2.14–2.19)	2.05 (2.03–2.08)
Preterm (<37 weeks)	1.54 (1.52–1.58)	1.51 (1.49–1.54)
Term (37+ weeks)	1.86 (1.84–1.88)	1.73 (1.72–1.75)
Maths		
Overall	2.42 (2.40–2.44)	2.29 (2.27–2.31)
Males	2.31 (2.28–2.33)	2.21 (2.18–2.32)
Females	2.54 (2.51–2.58)	2.41 (2.38–2.45)
Preterm (<37 weeks)	1.98 (1.94–2.03)	1.94 (1.89–1.98)
Term (37+ weeks)	2.40 (2.37–2.43)	2.28 (2.25–2.31)
Key Stage 2		
English		
Overall	2.05 (2.03–2.07)	1.94 (1.92–1.95)
Males	1.90 (1.88–1.92)	1.83 (1.81–1.85)
Females	2.22 (2.19–2.25)	2.13 (2.10–2.16)
Preterm (<37 weeks)	1.75 (1.71–1.79)	1.72 (1.68–1.76)
Term (37+ weeks)	2.10 (2.07–2.12)	1.97 (1.95–2.00)
Maths		
Overall	2.16 (2.15–2.18)	2.08 (2.07–2.10)
Males	2.14 (2.12–2.16)	2.06 (2.04–2.09)
Females	2.19 (2.16–2.22)	2.11 (2.08–2.14)
Preterm (<37 weeks)	1.93 (1.89–1.97)	1.90 (1.86–1.95)
Term (37+ weeks)	2.16 (2.14–2.19)	2.08 (2.06–2.11)

Adjusted models controlled for sex at birth, birth month, year of birth, neighbourhood deprivation level, region of residence, ethnicity and recorded eligibility for free school meals.

EYFSP, Early Years Foundation Stage Profile; ND, neurodisability; RR, risk ratio.

### Secondary analysis

Educational attainment varied considerably among children with different neurodisability-associated conditions (online supplemental table S6). In Maths assessments throughout primary school, attainment was highest among children with perinatal conditions, with 55%–60% achieving expected levels. This was followed by children with epilepsy (34%–38%), cerebral palsy (27%–36%) and neurodevelopmental disorders (24%–25%), while the lowest attainment was observed in children with Down syndrome (1%–4%; figure 1b). A similar pattern was observed in English assessments (online supplemental figure S4). Patterns of non-participation followed those seen in the overall neurodisability group, increasing by KS2. Notably, 97% of children with Down syndrome were not assessed in KS2, representing the highest rate of non-participation, whereas 19% of children with perinatal conditions were not assessed, indicating the highest participation rate among the neurodisability subgroups (online supplemental table S6).

Of the 51 289 children with neurodisability, 34 848 (67.9%) completed all three Maths and English assessments, compared with 91.9% of peers. Among children with neurodisability, those completing all assessments were more likely to meet expected levels at EYFSP, KS1 and KS2 than those missing at least one assessment (online supplemental table S7), though attainment remained lower than among unaffected peers.

## Discussion

In this national study of over 2 million children, fewer than half of those with neurodisability met expected attainment standards in Maths and English across primary school. While SEND provision, particularly EHCPs, increased over time, many children diagnosed before school entry were not identified early for support. The persistence of large attainment gaps suggests current provision is insufficient and highlights the opportunity to use clinical data to identify educational needs earlier and ensure appropriate support from school entry.

Children with neurodisability were over twice as likely to fail all Maths assessments and 1.7 times more likely to fail all English assessments. Non-participation in assessments increased substantially between KS1 and KS2, becoming the main reason for not achieving expected levels of attainment at the end of primary school. Attainment outcomes varied significantly by neurodisability subgroup. Children with perinatal conditions had the best attainment outcomes among children with neurodisability, with over 55% of them achieving expected levels in all primary school Maths assessments. The lowest attainment was observed among children with Down syndrome, where under 5% of children achieved expected levels every primary school assessment.

The increased relative risk of low attainment observed among females with neurodisability compared with unaffected peers is notable. This may reflect diagnostic differences, as neurodevelopmental conditions in females are often less overt and therefore under-recognised in milder forms.^20^ Consequently, diagnosed females may represent a more severely affected subgroup with higher unmet needs. The risk of low attainment was higher among term-born children, reflecting both stronger contrasts with typically higher-performing peers and the independent impact of neurodisability beyond prematurity.

A key strength of this study is the use of population-level linked health and education datasets across complete birth cohorts. The coverage of both the HES and NPD allowed us to follow children longitudinally across primary school and to examine outcomes for relatively rare neurodisability subgroups with high statistical precision. More than 95% of children enrolled in Reception remained enrolled through Year 6, ensuring near-complete follow-up prior to COVID-19-related disruptions. This represents the most comprehensive population-level examination of educational attainment in children with neurodisability in England to date.

Several methodological limitations should be considered. To ensure high-quality linkage between health and education records, children from multiple or twin births were excluded. As multiples have increased risks of prematurity and neurodisability,^21^ their exclusion may slightly underestimate neurodisability prevalence and attenuate attainment disparities. However, they represented less than 0.3% of the cohort (online supplemental figure S1).

Further, relying on hospital admissions before age 5 to identify neurodisability introduces important limitations. This approach preferentially captures children with more severe or medically complex conditions that require early secondary-care involvement, while under-ascertaining neurodevelopmental conditions typically diagnosed later in childhood or managed primarily in community, educational or mental health settings. Under-represented conditions include ADHD, ASD, conduct disorders and specific learning disabilities, which are often diagnosed after school entry.^3^ Consequently, children with milder forms of neurodisability, or neurodevelopmental disorders typically diagnosed later in childhood, may have been included in the comparison group. This exposure misclassification may lead to underestimation of neurodisability prevalence and attainment disparities observed in this study. Despite the likely under ascertainment of certain conditions, the prevalence of recorded neurodisability in our cohort was highly stable over time (2.1%–2.3%). This supports the validity of our code list in consistently identifying a robust and clinically meaningful subset of children with neurodisability.

Interpretation of attainment outcomes is further constrained by differences in assessment methodology across primary school stages. EYFSP and KS1 outcomes are teacher-assessed, while KS2 assessments are externally marked, potentially introducing variability related to school-level marking practices or teacher perceptions. However, these remain the only primary school outcome measures available in the NPD. Future studies should examine more recent cohorts to assess whether attainment gaps differ in later years, given that assessment structures have been revised since the study period.^19 22^

Our findings indicate that national standardised assessments do not sufficiently capture educational progress for many children with neurodisability. Over 30% of children with neurodisability do not participate in KS2 assessments, meaning they are officially recorded as not achieving expected attainment levels despite potentially making significant progress in areas not measured by national assessments. In state-funded schools in England, children working below national curriculum standards are typically assessed under the Engagement Model,^23^ which takes a child-centred approach, focusing on developmental progress rather than curriculum benchmarks. However, these data are not recorded in the NPD and therefore cannot be evaluated at the population level.^23^ Integrating these outcomes into national datasets is essential for representing long-term educational progress in children with neurodisability on a population level.

We demonstrate that children who are likely to face functional impairments from neurodisability can be identified in hospital records before school entry, presenting a valuable opportunity for early intervention. Only a third of children with neurodisability was deemed ‘school ready’ by the GLD at the end of Reception, yet nearly all (99%, online supplemental table S4) transitioned to Year 1 the following term. Given these children are already known to health services, there is clear potential for better coordination between health and education to ensure that preidentified support needs are in place on starting school. Although three-quarters of children with neurodisability eventually receive SEND provision, support is frequently delayed and inconsistently applied.^3^ Greater integration between healthcare and educational assessment pathways could facilitate more timely initiation of provision.

To maximise the impact of early intervention, it is important to recognise that educational outcomes are shaped by intersecting structural disadvantages. Although our models were stratified by sex and adjusted for birth season, birth year, neighbourhood deprivation level, region of residence, ethnicity and recorded eligibility for free school meals, we did not explore within-group inequalities among children with neurodisability. Educational outcomes are likely shaped by intersecting structural disadvantages, and future research should examine their interactions with neurodisability to identify subgroups at highest risk of poor attainment and unmet need.

Education is a key social determinant of outcomes throughout adulthood. Thus, further research is needed to follow these children through secondary school and beyond, as children with neurodisability are more likely to leave school before 16, face exclusion and be not in education, employment or training.^5 24–26^

Our study adds to a limited body of evidence on neurodisability in England. We demonstrate that children with neurodisability are less likely to achieve expected levels of attainment in primary school compared with their peers. The progress made by these children is currently inadequately captured in population-level data. Supporting children with neurodisability will improve their participation in society and transition to independence.^27^

## Supplementary material

10.1136/archdischild-2025-329224 online supplemental file 1

10.1136/archdischild-2025-329224 online supplemental file 2

## Data Availability

All data relevant to the study are included in the article or uploaded as supplementary information.
