# Supplementary material for: Educational attainment among primary school children with neurodisability: a population-based cohort study using linked education and health data from England
Source: Arch Dis Child. 2026 Mar 22;111(8):e329224. doi: 10.1136/archdischild-2025-329224 (PMC13422109; doi:10.1136/archdischild-2025-329224)
Supplement: online supplemental file 2 [file archdischild-111-8-s002.pdf]

Educational attainment among primary school children with neurodisability: A population-based cohort study using linked education and health data from England.

National Pupil Database: Information on all children in state schools in England

echild Database

Hospital Episode Statistics: Details of all publicly-funded hospital activity in England

Study Cohort:

- Born in England in publicly-funded hospitals between 1st September 2023 and 31st August 2008
- Enrolled in state-funded primary school (Reception, age 4/5)

Neurodisability first recorded in hospital admissions data before Reception (age 4/5)

Outcomes: Educational attainment in Maths and English at Reception (age 4/5), Key Stage 1 (age 6/7) and Key Stage 2 (age 10/11)

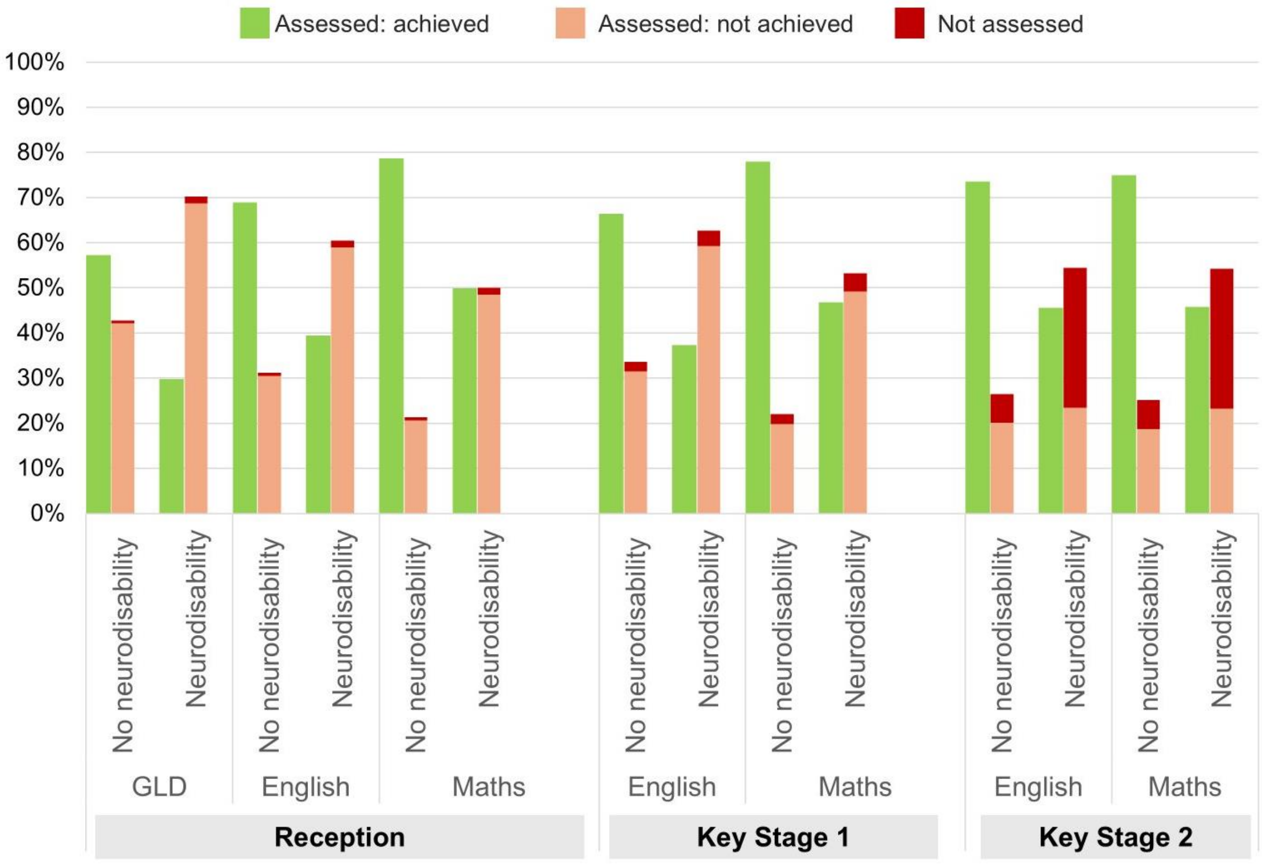

BMJ Group
RCPCH Royal College of Paediatrics and Child Health Leading the way in Children's Health
adc.bmj.com fn.bmj.com ep.bmj.com
Archives of Disease in Childhood
